# Targeting HMGCS1 restores chemotherapy sensitivity in acute myeloid leukemia

**DOI:** 10.1097/BS9.0000000000000192

**Published:** 2024-07-10

**Authors:** Cheng Zhou, Jue Li, Xiaofan Sun, Liang Zhao, Huien Zhan, Hui Liang, Peng Fang, Tuo Zhang, Qiongzhi He, Juan Du, Hui Zeng

**Affiliations:** a Department of Hematology, The First Affiliated Hospital of Jinan University, Guangzhou, Guangdong 510630, China; b Department of Hematology, Xiangya Hospital, Central South University, Changsha, Hunan 410008, China; c Genomic Core, Weill Cornell Medical College, New York, NY 10021, USA; d Geneplus-Beijing Institute, Beijing 102206, China

**Keywords:** Chemotherapy sensitivity, HMGCS1, MAPK pathway, Relapsed and refractory AML, Targeted therapy

## Abstract

Acute myeloid leukemia (AML) is a common hematological malignancy with overall poor prognosis. Exploring novel targets is urgent and necessary to improve the clinical outcome of relapsed and refractory (RR) AML patients. Through clinical specimens, animal models and cell-level studies, we explored the specific mechanism of 3-hydroxy-3-methylglutaryl coenzyme A synthase 1 (HMGCS1) in AML and the mechanism of targeting HMGCS1 to attenuate cell proliferation, increase chemotherapy sensitivity and improve the occurrence and development of AML. Here, we reveal that HMGCS1 is overexpressed in RR patients and negatively related to overall survival (OS). Knocking out HMGCS1 in AML cells attenuated cell proliferation and increased chemotherapy sensitivity, while stable overexpression of HMGCS1 had the opposite effects. Mechanistically, we identified that knockout of HMGCS1 suppressed mitogen-activated protein kinase (MAPK) pathway activity, while overexpression of HMGCS1 could remarkably enhance the pathway. U0126, a MEK1 inhibitor, offset the effects of HMGCS1 overexpression, indicating that HMGCS1 promotes RR AML through the MAPK pathway. Further, we verified that hymeglusin, a specific inhibitor of HMGCS1, decreases cell growth both in AML cell lines and primary bone marrow cells of AML patients. Furthermore, combination of hymeglusin and the common chemotherapeutic drug cytarabine and adriamycin (ADR) had synergistic toxic effects on AML cells. Our study demonstrates the important role of HMGCS1 in AML, and targeting this protein is promising for the treatment of RR AML.

## 1. INTRODUCTION

Acute myeloid leukemia (AML) is a malignant clonal hematopoietic disease characterized by abnormal aggregation of immature blasts in the bone marrow and hindrance to the generation of normal blood cells. AML is one of the most common leukemia types in adults. Despite rapid progress in the development of novel drugs and approaches, highly effective treatments with minimal side effects remain absent, particularly for relapsed and refractory (RR) AML patients. The outcome of RR AML patients is extremely poor with a 3-year OS estimated to be less than 10%.^1,2^

As an emerging hallmark of cancer, cell metabolism has been recognized to play a significant role in cancer pathogenesis.^3^ Similar to solid tumors, leukemia has distinct metabolic profiles. A total of 45 metabolic pathways were altered in AML patients, and among these, glucose and lipid metabolic alterations were remarkably associated with survival.^4^ The aberrant metabolites mainly include lipids, lactate, and glucose.^5^ Cholesterol metabolism is dysregulated in AML, and clinicians have recognized that hypocholesterolemia is common in newly diagnosed AML patients,^6^ which is likely the result of a vast demand of cholesterol to satisfy rapid growth. Consistent with this idea, AML cells synthesize more cholesterol and import more cholesterol than normal cells.^7^ In addition, when exposed to chemotherapy in vitro, AML cells demonstrated abnormally increased cholesterol levels, which mainly derive from de novo synthesis.^8^ Based on these data, interrupting the increase in protective cholesterol by suppressing its synthesis appears to be a plausible strategy for sensitizing AML cells to treatment.

De novo cholesterol synthesis starts with the synthesis of acetoacetyl-CoA from acetyl-CoA. 3-Hydroxy-3-methylglutaryl coenzyme A (HMG-CoA) is then produced by the condensation of acetoacetyl-CoA and acetyl-CoA, which is catalyzed by HMG-CoA synthase 1 (HMGCS1). The next step is the first commitment step toward the conversion of HMG-CoA to mevalonate (MVA) by HMG-CoA reductase (HMGCR). MVA is further converted into sterols and isoprenoids, such as farnesyl pyrophosphate (FPP), geranylgeranyl pyrophosphate (GGPP), and cholesterol through a series of enzymatic steps,^9^ which have been shown to be crucial for tumor growth.^10^

HMGCR, the rate limiting enzyme, has attracted extensive attention. As a specific inhibitor of HMGCR, statins are extensively used as a cholesterol-lowering agents.^11^ Although several phase 1/2 clinical trials have demonstrated that statins can improve the efficacy of standard therapy in AML,^12,13^ leukemia cells actually display a heterogenous response to statins.^14,15^ Thus, enzymes other than HMGCR can be potential targets, and increasing studies are focusing on enzymes that control cholesterol synthesis.^16^ We approached this question by profiling gene expression differences between complete remission (CR) and RR patients using RNA-seq. Among the genes differentially expressed between these 2 groups, we found that HMGCS1 was significantly upregulated in RR AML patients. As an upstream enzyme in the de novo cholesterol synthesis pathway, HMGCS1 has been reported to enhance tumor growth in melanoma.^17^ HMGCS1 was also upregulated in multiple myeloma (MM) cells insensitive to statins^18^ and AML cells treated with statins.^19^ However, the specific role of HMGCS1 in AML has not been reported yet.

Here, we demonstrate that HMGCS1 not only promotes leukemia cell proliferation but also impairs the efficacy of standard anti-leukemia regimens in AML. HMGCS1 upregulation activates the mitogen-activated protein kinase (MAPK) pathway. Inhibiting HMGCS1 by hymeglusin could suppress the growth of AML cells and enhance their chemosensitivity. Importantly, hymeglusin synergized with the chemotherapeutic drugs adriamycin (ADR) and cytarabine (Ara-C) to achieve efficient inhibition of AML cell growth. Additionally, a xenograft nude mouse model was established and demonstrated that HMGCS1 overexpression (OE) promotes AML tumor growth. Taken together, our findings identified HMGCS1 as a potential target and its inhibitor hymeglusin as a promising drug candidate for AML treatment.

## 2. MATERIALS AND METHODS

### 2.1. Ethics statement

This study conformed to the guidelines approved by the Ethics Committee of Xiangya Hospital, Central South University. Signed informed consent was obtained from all participants or their guardians (approval number: 201912530). Animals were housed and experiments were conducted at the specific pathogen free (SPF) grade animal facility Experiment Animal Center at Central South University. All animal experiments were approved by the Institutional Animal Care and Use Committee of Central South University-Xiangya Hospital (IACUC-CSU-XH) and performed according to the approved protocols by IACUC-CSU-XH. All efforts were made to minimize the suffering of the included animals.

### 2.2. Cell lines and culture conditions

The HL-60, THP-1, KG-1, and HL-60 Ara-C cell lines were obtained from the Cell Resource Center (Xiangya Medical College, Central South University, Hunan, China) and cultured in RPMI-1640 medium (Gibco, Carlsbad) supplemented with 10% fetal bovine serum (Gibco, Grand Island) and a 1% antibiotic solution of penicillin and streptomycin (Corning Inc.) in a humidified atmosphere containing 5% CO_2_ at 37°C.

### 2.3. Patient characteristics

Lipid level data from 257 CR and 78 refractory and relapsed (RR) patients without clinical usage of lipid lowering agents were collected to compare lipid profiles. The characteristics of these patients are included in Tables 1 and 2. Another collection of 21 CR and 21 RR AML samples was used for RNA-seq. The patient characteristics of these bone marrow samples were described previously. A total of 87 bone marrow samples were collected from AML patients to validate the HMGCS1 expression level, and 9 samples from healthy hematopoietic stem cell transplant donors were obtained as controls. The characteristics of these 87 patients are included in Tables 3 and 4, and Table 5 lists the characteristics of patients whose samples were included in vitro assays. All patients were treated at the Xiangya Hospital of Central South University, Hunan, China. Mononuclear cells (MNCs) were obtained by density centrifugation over a Ficoll-Paque gradient (Eppendorf, Hamburg, Germany) and stored at −80°C. The experiments were approved by the Medical Ethics Committee of Xiangya Hospital, Central South University. The diagnosis and classification of AML were based on the 2016 World Health Organization (WHO) criteria.

**Table 1:** Brief patient information of 257 CR and 78 RR AML bone marrow samples for lipid level comparison.

Groups	Complete remission (257)	Relapsed/refractory (78)
Average age (y)	42.47 (13–71)	48.65 (14–75)
Gender	135 (52.53%)/140 (47.47%)	37 (47.44%)/41 (52.56%)
Gene mutation	109 (42.41%)	49 (62.82%)
Chromosomal abnormality	52 (20.23%)	27 (34.62%)
Favorable	35 (13.6%)	16 (20.5%)
Intermediate	165 (64.2%)	36 (46.2%)
Adverse	57 (22.2%)	26 (33.3%)

AML = acute myeloid leukemia, CR = complete remission, RR = relapsed and refractory.

Genetic-risk groups were defined as favorable/intermediate/adverse using the ELN-2017 classification system.

**Table 2:** FAB classification, gene mutations and chromosomal abnormality of 257 CR and 78 RR AML bone marrow samples for lipid level comparison.

Sample classification	n (%)
AML FAB classification	
M1	17 (5.1)
M2	166 (49.6)
M3	8 (2.4)
M4	36 (10.7)
M5	63 (18.8)
M6	5 (1.5)
Undetermined	40 (11.9)
Gene mutation	
WT1	11 (3.3)
ETO	25 (7.5)
FLT3-ITD	29 (8.7)
CEBPA	46 (13.7)
NPM1	31 (9.3)
TP53	2 (0.6)
CKit	7 (2.1)
IDH1	6 (1.8)
IDH2	8 (2.4)
DNMT3A	17 (5.1)
TET2	12 (3.45)
HOX11	6 (1.8)
MLL	3 (0.9)
JAK2	6 (1.8)
NRAS	19 (5.6)
RUNX1	2 (0.6)
SETBP1	4 (1.2)
PHF6	10 (3.0)
BCOR	2 (0.6)
PTPN11	2 (0.6)
GATA2	2 (0.6)
Chromosomal abnormality	
t (8;21)	12 (3.4)
t 10;11)	8 (2.4)
t (11;13)	10 (3.0)
t (2;4)	4 (1.2)
Del (9)	11 (3.3)
9q-	4 (1.2)
+(8)	7 (2.1)
−7	1 (0.3)
5q-	1 (0.3)
Complex karyotype	19 (5.6)

AML = acute myeloid leukemia, CR = complete remission, FAB = Fench–American–British classification systems, RR = relapsed and refractory, WHO = World Health Organization.

All AML patients were identified by 2016 WHO criteria.

**Table 3:** Brief patient information of 87 AML bone marrow samples for validation of the expression level of HMGCS1.

Groups	Relapsed/refractory (46)	Complete remission (41)
Average ages (y)	44.87 (15–64)	40.5 (15–86)
Gender	27 (58.7%)/19 (50%)	24 (58.5%)/17 (41.5%)
Gene mutation	28 (60.8%)	18 (43.9%)
Chromosomal abnormality	17(36.9%)	12 (29.2%)
Favorable	5 (10.87%	9 (21.95%)
Intermediate	26 (56.53%)	25 (60.98%)
Adverse	15 (32.6%)	7 (17.07%)

AML = acute myeloid leukemia, HMGCS1 = 3-hydroxy-3-methylglutaryl coenzyme A synthase 1.

**Table 4:** FAB classification, gene mutation and chromosomal abnormality of 87 AML bone marrow samples for validation of expression level of HMGCS1.

Sample classification	n (%)
AML FAB classification	
M1	4 (4.6)
M2	35 (40.2)
M3	3 (3.5)
M4	12 (13.8)
M5	15 (17.2)
M6	1 (1.1)
Undetermined	17 (19.6)
Gene mutation	
WT1	7 (8.0)
ETO	9 (10.3)
FLT-ITD	14 (16.1)
CEBPA	9 (10.3)
NPM1	10 (11.5)
CSF3R	1 (1.1)
KDM6A	1 (1.1)
DNMT3A	1 (1.1)
TET2	3 (3.4)
MLL/AF9	1 (1.1)
NF1	1 (1.1)
Chromosomal abnormality	
t (8;21)	3 (3.4)
t (9;11)	1 (1.1)
t (2;4)	1 (1.1)

AML = acute myeloid leukemia, FAB = Fench–American–British classification systems, HMGCS1 = 3-hydroxy-3-methylglutaryl coenzyme A synthase 1.

**Table 5:** Brief patient information of 6 AML primary cells used for in vitro assays.

De novo AML patients	Ages (y)	Gender	Risk stratification	FAB classification
#1	57	Man	Intermediate	M2a
#2	59	Female	Intermediate	M2
#3	54	Female	Favorable	M2a
#4	30	Female	Adverse	M5
#5	59	Man	Favorable	Undetermined
#6	59	Female	Adverse	M4

AML = acute myeloid leukemia, FAB = Fench–American–British classification systems.

### 2.4. Construction of lentiviral vectors

Stable knockout (KO) of endogenous human HMGCS1 was achieved by using lentiviral vector GV392 harboring the sgRNA construct TGCACTGAGGTAGCACTGTA (#1) or GGATATTCAGATAGCATATC (#2). An HMGCS1 OE plasmid was cloned into the GV208 lentiviral vector backbone. Both were then transfected into 293T cells to produce lentiviruses.

### 2.5. Lentiviral infection of cell lines

Lentiviruses were purchased from the company GeneChem. HL-60 and THP-1 cells were resuspended in enhanced infection solution (Catalog No. REVG0002). A total of 5 × 10^4^ cells/mL were seeded in 96-well plates (3 sub-wells for each cell line). HMGCS1 OE, and HMGCS1 KO lentiviruses and their respective control lentiviruses at a titer of 1 × 10^8^ TU/mL were added to the corresponding wells (multiplicity of infection [MOI], 20–50). Stable cells were selected with puromycin at a concentration of 2 µg/mL. The HMGCS1 KO efficiency was detected by western blot (WB) and reverse transcription-polymerase chain reaction (RT-PCR).

### 2.6. Cytotoxicity assay

To evaluate the cell response to different drugs, cell viability was determined by a Cell Counting Kit (Dojin Laboratories, Kumamoto, Japan) after treatment. AML cell lines (HL-60 and THP-1) or primary cells (HSPC and leukemia cells from a patient) were seeded in 96-well culture plates at the density of 5 × 10^4^ cells/mL and incubated with (*R*, *R*)-hymeglusin (Abcam, Boston, Massachusetts, ab144274), adriamycin (Medchemexpress, HY-15142), and U0126 (Medchemexpress, HY-12031). After certain times, 10 μL CCK-8 solution was added to each well for a 3-hour culture at 37°C. Absorbance was measured by a spectrophotometer (Bio Tek Instruments) at the wavelength of 450 nm.

### 2.7. Quantitative RT-PCR

To detect the expression of HMGCS1 in the different clinical samples, RNA and protein were isolated from the MNCs of all patients and healthy donors. Total RNA was isolated from 1 × 10^6^ cells using TRIzol reagent (TaKaRa, Otus, Japan). Total cDNA was prepared using a 2-step reverse transcription kit (TaKaRa). Primers for real-time PCR were obtained from Integrated DNA Technologies. The primers were (HMGCS1/forward) 5′-CTTCAGGTTCTGCTGCTGTG-3′, (HMGCS1/reverse) 5′-CAGAAGAACTTACGCTCGGC-3′; (glyceraldehyde-3-phosphate dehydrogenase [GAPDH]/forward) 5′-CTTTGTCAAGCTCATTTCCTGG-3′; (GAPDH/reverse) 5′-TCTTCCTCTTGTGCTCTTGC-3′. Reactions were run on an Applied Biosystems Prism machine using ABI StepOnePlus (Applied Biosystems, Foster City, California). The transcript levels for the genes of interest were normalized to the GAPDH transcript levels.

### 2.8. Western blot analysis

Equal amounts of protein were separated by 10% sodium dodecyl sulfate-polyacrylamide gel electrophoresis (SDS-PAGE) and then electrophoretically transferred to polyvinylidene fluoride (PVDF) membranes (Millipore, Billerica, Massachusetts). The membranes were incubated with PBS-T containing 5% BSA (Bio Sharp Sigma A-4612, Guangzhou, China) for an hour at room temperature and then with primary antibodies overnight at 4°C. After incubation with secondary antibodies, the protein bands were detected with a Chei DocTMMP imaging system (BioRad). The antibody dilutions used were as follows: HMGCS1 1:2,000 (Abcam, ab87246, USA), ERK1/2 1:2,000 (Cell Signaling Technology, #9102, USA), MEK1 1:2,000 (CST, #8727, USA), Phospho-ERK1/2 1:2,000 (CST, #9488, USA), Phospho-MEK1 1:2,000 (CST, #98195, USA), and GAPDH 1:2,000 (Proteintech, SA00001-2, Wuhan, China).

### 2.9. 5-Ethynyl-2’-deoxyuridine (EdU)^+^ staining

An EdU-based cell proliferation kit, Cell-Light EdU Apollo567 In Vitro Kit (Ribobio, Guangzhou, China), was used to measure DNA synthesis according to the manufacturer’s instructions. All steps were performed at room temperature.

### 2.10. Apoptosis assessment

A total of 1 × 10^6^ cells were treated with hymeglusin and/or ADR, and they were collected after a 24-hour incubation and washed twice with cold PBS. Subsequently, the cells were resuspended in 100 μL binding buffer, stained with Annexin-V and 7-AAD at a dilution of 1:20 and 1:10 (Becton Dickinson, Los Angeles, California), incubated in the dark on ice for 15 minutes, and subjected to flow cytometry analysis (Becton Dickinson).

### 2.11. Cell cycle analysis

The cell cycle was analyzed using a Cell Cycle Staining Kit (Liankebio, China). As described previously, 10^6^ cells were seeded in 6-well plates for 24 hours. The cells were harvested, washed twice with 1× PBS, and incubated with 1 mL of DNA staining solution containing propidium iodide and 10 μL of permeabilization solution in the dark for 30 minutes at room temperature. The cell cycle status was then analyzed using a flow cytometer (Becton Dickinson).

### 2.12. RNA sequencing

RNA was extracted from pooled bone marrow samples from either CR (n = 21) or relapse/refractory (n = 21) patients. RNA sample quality was analyzed, and cDNA libraries were prepared and sequenced using BGI RNA-seq service (BGI, Shenzhen, China). Each library was sequenced on a HiSeq4000 (Illumina) sequencer using single reads. Gene expression levels were measured in fragments per kilobase million (FPKM) using RNA-Seq by expectation-maximization (RSEM). Differentially expressed genes were identified with an analysis method based on Poisson distribution. For RNA-seq of stable cell lines, 1 to 2 million cells were collected for each replicate, and 3 replicates in each group were used to construct sequencing libraries. Sequencing and analysis were performed by Biomarker Technologies (Biomarker, Wuhan, China) and IGE Technology (IGE, Guangzhou, China).

### 2.13. Xenograft model

A total of 24 nude mice (nu/nu, female 4–6 weeks old) were randomly allocated to 2 groups and injected subcutaneously with 3 × 10^6^ THP-1 Vector/OE cells (n = 7 for each group) and Ctrl/KO #2 (n = 5 for each group). Tumor sizes were measured every 2 days using a Vernier caliper. Tumor growth was recorded by measurement of three perpendicular diameters using the formula (min^2^ × max)/2. The mice were sacrificed by carbon dioxide asphyxiation followed by cervical dislocation at day 30, and the tumors were then harvested and weighed. Statistical analysis was performed using 2-way analysis of variance (ANOVA) for tumor volume and with 2-tailed Student *t* test for tumor weight. Animals were housed and experiments were conducted at the SPF grade animal facility Experiment Animal Center at Central South University. All animal experiments were approved by the Institutional Animal Care and Use Committee of Central South University- Xiangya Hospital (IACUC-CSU-XH) and performed according to the approved protocols by IACUC-CSU-XH.

### 2.14. Statistical analysis

In studies where statistical analyses were performed, differences among these groups were tested by Student *t* test or 1-way ANOVA as appropriate. *P* values ≤.05 were considered significant. Two-way ANOVA was used for tumor volume. The data are represented as the mean ± SD. *P < .05, **P < .01, and ***P < .001.

## 3. RESULTS

### 3.1. HMGCS1 expression levels correlate with AML status and therapeutic effects

**Figure 1.**
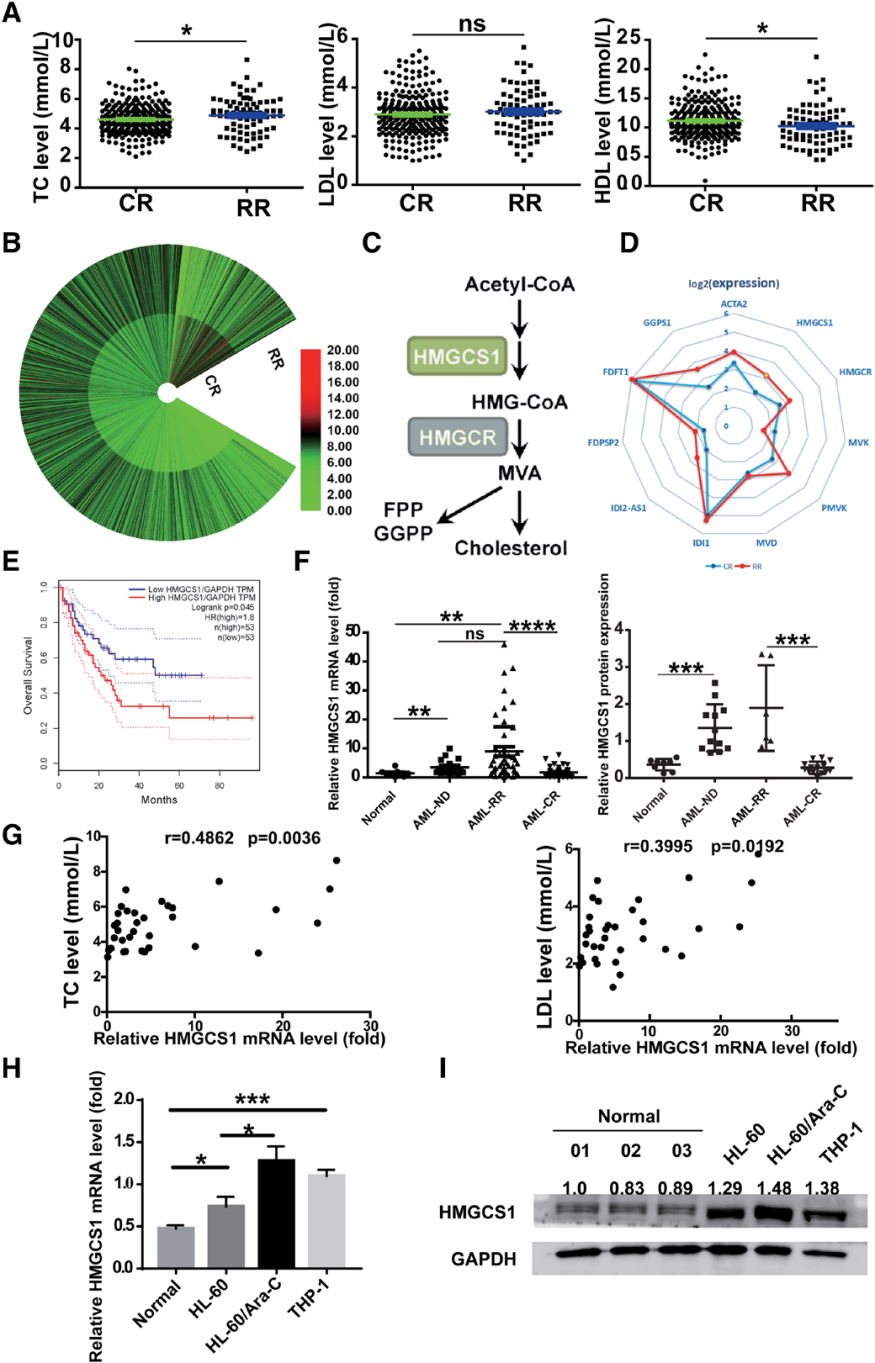
Aberrant lipids level and HMGCS1 level in clinical AML samples. (A) The serum lipid level, including TC (left), LDL (middle), and HDL (right) in 257 CR and 78 RR patients without clinical usage of lipid lowering agents. (B) Gene expression profile of CR and RR AML patients by RNA-Seq. (C) Illustration of de novo cholesterol synthesis pathway. (D) Expressions of key genes in the de novo cholesterol synthesis pathway in (B). (E) Survival analysis of AML patients based on gene expression level of HMGCS1. This curve was derived from GEPIA database (http://gepia2.cancer-pku.cn/#survival). (F) Different HMGCS1 mRNA level (left) and representative western blot quantification of protein level (right) between normal, CR and RR group. Characteristics of these patients are included in Table 3. (G) Correlation between serum TC (left) and LDL (right) level and HMGCS1 mRNA level in AML patients. (H) HMGCS1 mRNA level in normal bone marrow cells, AML WT cell lines (HL-60, THP-1) and HL-60/Ara-C cells (Ara-C resistant HL-60 cells). (I) Protein level of HMGCS1 in normal bone marrow cells, HL-60, HL-60 Ara-C, and THP-1 cells detected by Western blot. *P < .05, **P < .01, ***P < .001, ****P < .0001. AML = acute myeloid leukemia, CR = complete remission, FPP = farnesyl pyrophosphate, GGPP = geranylgeranyl pyrophosphate, HDL = high-density lipoprotein, HMGCS1 = hydroxy-3-methylglutaryl coenzyme A synthase 1, LDL = low-density lipoprotein, RR = refractory and relapse, TC = total cholesterol.

Previous reports^6,8^ have shown that the serum lipid levels in AML patients were closely related to disease status. To verify the correlation between serum lipid levels and AML disease status, we investigated the serum lipid levels in clinical AML patients, including CR (n = 257) and RR (n = 78) patients. Significantly higher levels of total cholesterol (TC) and total triglyceride (TG) (Figure S1A, http://links.lww.com/BS/A98) were detected in AML RR patients than in CR patients (**Fig. 1A**, left). Interestingly, the low-density lipoprotein (LDL) level in AML RR patients was comparable to that in CR patients (**Fig. 1A**, middle), while high-density lipoprotein (HDL) was lower in AML RR patients than in CR patients (**Fig. 1A**, right). Gene expression patterns from RNA-Seq data showed a great difference between the CR and RR groups (**Fig. 1B**). De novo cholesterol synthesis pathway genes including ACAT2, HMGCS1, HMGCR, FDPS, IDI2, PMVK, and GGPSS were activated in the RR group (**Fig. 1C and D**). We focused on HMGCS1 as it was an upstream enzyme in the cholesterol biosynthesis pathway, and it was expressed 2-fold higher in the RR group than in the CR group. GEPIA (a web server for cancer and normal gene expression profiling and interactive analyses) was utilized to analyze the clinical influence of HMGCS1 on AML. Survival analysis based on gene expression levels demonstrated that AML patients with higher HMGCS1 expression exhibited shorter OS (**Fig. 1E**, *P* = .045).

By analyzing the HMGCS1 expression levels in MNCs of 87 AML bone marrow samples, we found that the mRNA and protein levels of HMGCS1 were significantly higher in the RR compared with CR group (**Fig. 1F**). Moreover, the HMGCS1 level was positively correlated with the TC and LDL levels (**Fig. 1G**). Furthermore, the HMGCS1 mRNA level was higher in AML cell lines (HL-60, THP-1) than in normal bone marrow cells. Ara-C and ADR-resistant HL-60 cells demonstrated the highest HMGCS1 mRNA level (**Fig. 1H** and Figure S1B, http://links.lww.com/BS/A98). The HMGCS1 protein levels in those cell lines demonstrated a similar trend as the mRNA levels. Interestingly, the HMGCS1 protein level in HL-60/Ara-C and HL-60/ADR were significantly higher compared to that in HL-60 cells (**Fig. 1I** and Figure S1C, http://links.lww.com/BS/A98). At the same time, the HMGCS1 protein and mRNA levels were significantly higher in THP-1 cells treated with ADR for 48 h (Figure S1D–E, http://links.lww.com/BS/A98). Collectively, these data suggest that HMGCS1 is upregulated in AML cell lines and higher HMGCS1 expression levels are positively correlated with RR status and poor survival in AML patients.

### 3.2. HMGCS1 promotes cell growth and reduces drug sensitivity in AML cells

**Figure 2.**
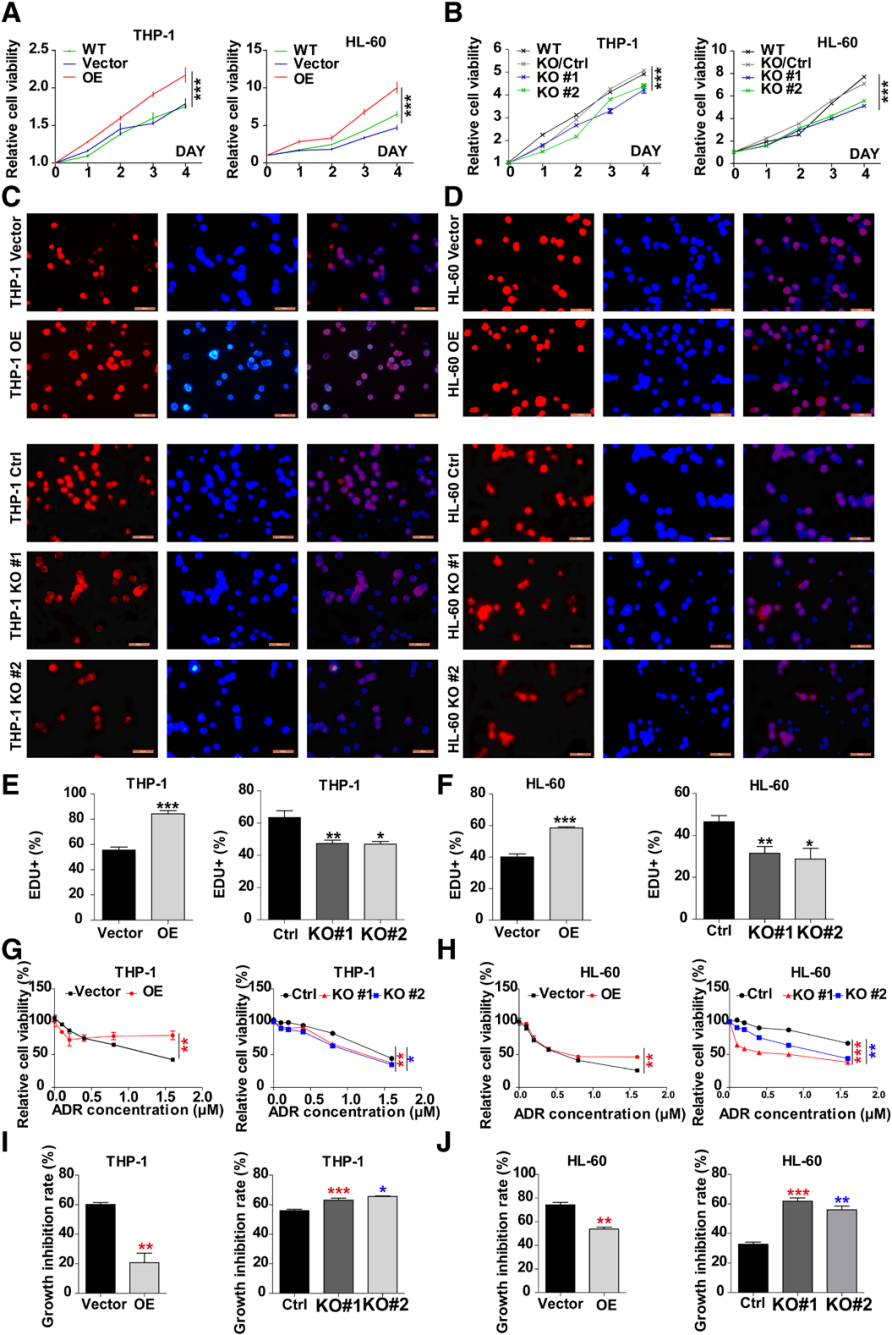
Cell proliferation and drug sensitivity of THP-1 and HL-60 stable cells. (A) Cell viability in HMGCS1 OE cells using CCK8 method. (B) Cell viability in HMGCS1 KO cells using CCK8 method. (C–D) Cell proliferation by EDU+ assay in HMGCS1 OE and KO THP-1 andHL-60 stable cell lines. (E–F) The statistical analysis of THP-1 cells in (C) and HL-60 cells in (D), representing the percentage of EDU+. (G–H) Relative cell viability in THP-1 and HL-60 and their HMGCS1 OE and KO stable cell lines treated with ADR. Cell viability was detected with CCK-8 kit 24 h after ADR treatment. (I–J) Statistical analysis of growth inhibition rate of THP-1 and HL-60 and their HMGCS1 OE and KO stable cell lines with 0.8 μM ADR treatment in (G–H). The *P* values were obtained by 2-way ANOVA. *P < .05, **P < .01, and ***P < .001. ADR = adriamycin, ANOVA = analysis of variance, EDU = 5-Ethynyl-2'-deoxyuridine, KO = knockout, OE = overexpression, WT = wild type.

We further explored the role of HMGCS1 in AML cell lines by establishing OE and HMGCS1 KO stable AML cell lines to examine their growth and drug sensitivity. AML cell lines overexpressing HMGCS1 THP-1/OE and HL-60/OE were established by lentivirus infection followed by drug selection, and the OE of HMGCS1 was confirmed. We established HMGCS1 KO stable THP-1 and HL-60 cell lines using CRISPR-CAS9 (Figure S2, http://links.lww.com/BS/A98). KO cell lines demonstrated over 70% reduction in the HMGCS1 expression level. Compared to their respective groups, the cell growth rate was significantly promoted in OE cells and inhibited in KO cells for both THP-1 and HL-60 cells (**Fig. 2A and B**, *P* < .0001). Consistent with the cell growth rate, the EDU^+^ labeling rate was also significantly higher in OE cells and lower in KO cells compared with their respective negative control (**Fig. 2C–F**). In addition, compared with control cells, OE cells were less susceptible to ADR, a drug commonly used that offers survival improvement for AML patients, while KO cells were more susceptible to ADR treatment (**Fig. 2G–J**).

### 3.3. HMGCS1 promotes AML tumor growth in a xenograft animal model

**Figure 3.**
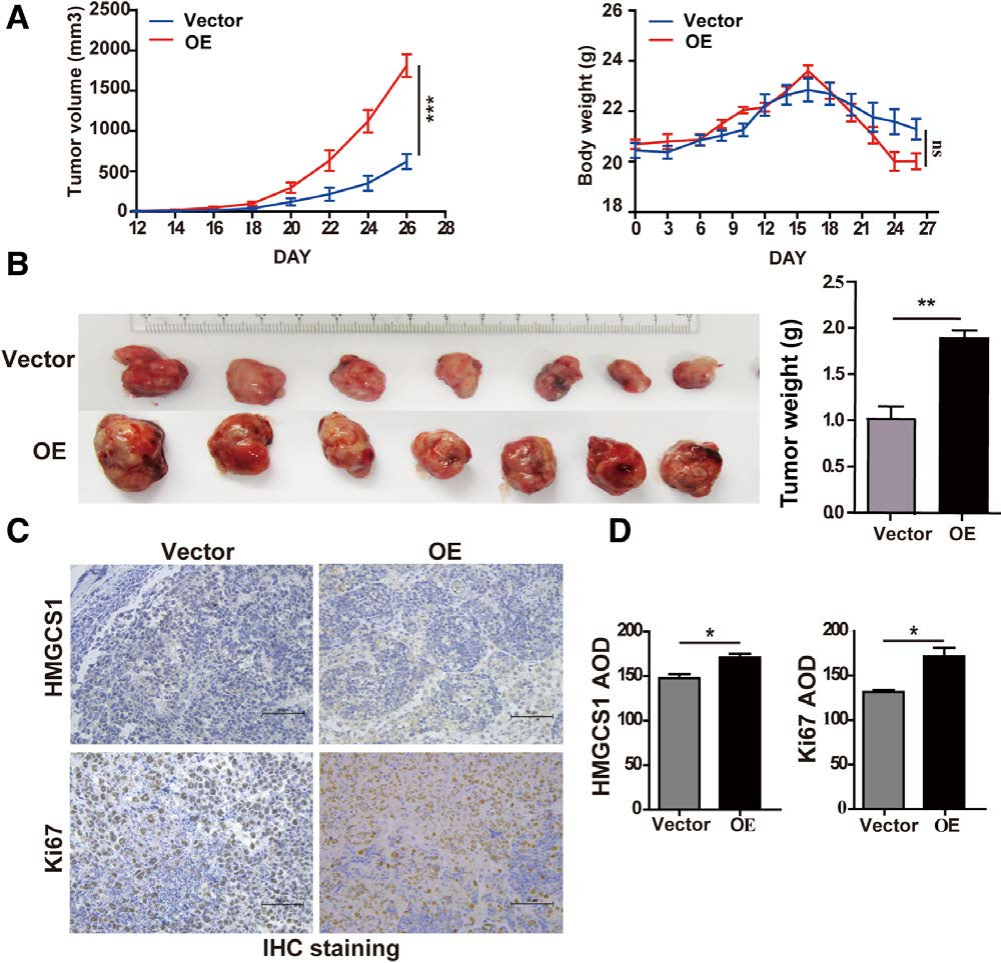
HMGCS1 could promote tumor growth in a xenograft nude mice model. (A) Tumor volume (left) and mouse body weight (right) of xenograft mice injected with THP-1 vector/OE cells (n = 7 for each group). (B) Tumor image (left) and weight (right) in (A). (C) Representative IHC staining images of HMGCS1 and Ki67 of xenograft tumor tissues. (D) The AOD of HMGCS1 (left) and Ki67 (right) in (C). Two-way ANOVA analysis was used for tumor volume. The data are represented as the mean ± SD. *P < .05, **P < .01, and ***P < .001. AOD = average optical density, ANOVA = analysis of variance, HMGCS1 = hydroxy-3-methylglutaryl coenzyme A synthase 1, IHC = immunohistochemistry, OE = overexpression, SD = standard deviation.

To investigate whether HMGCS1 promotes AML tumor growth, we established a xenograft model by injecting HMGCS1 OE or KO THP-1 cells subcutaneously into nude mice (**Fig. 3** and Figure S3, http://links.lww.com/BS/A98). OE of HMGCS1 could increase tumor growth rates (**Fig. 3A**, left, *P <* .0001) with minimal impact on mouse weight (**Fig. 3A**, right). Tumors harvested from xenograft nude mice injected with THP-1/OE cells had larger sizes and elevated weight compared to those derived from control vector stable cells (**Fig. 3B**, *P* = .0021). Immunohistochemistry (IHC) staining (**Fig. 3C**) demonstrated that the HMGCS1 expression was much higher in the OE group (**Fig. 3D**, left), which was positively correlated with the Ki67 level (**Fig. 3D**, right), an indicator of the proliferation index. Together, these data suggest that HMGCS1 promotes the growth of AML cells in vivo.

### 3.4. HMGCS1 activates the MAPK pathway in AML cells

**Figure 4.**
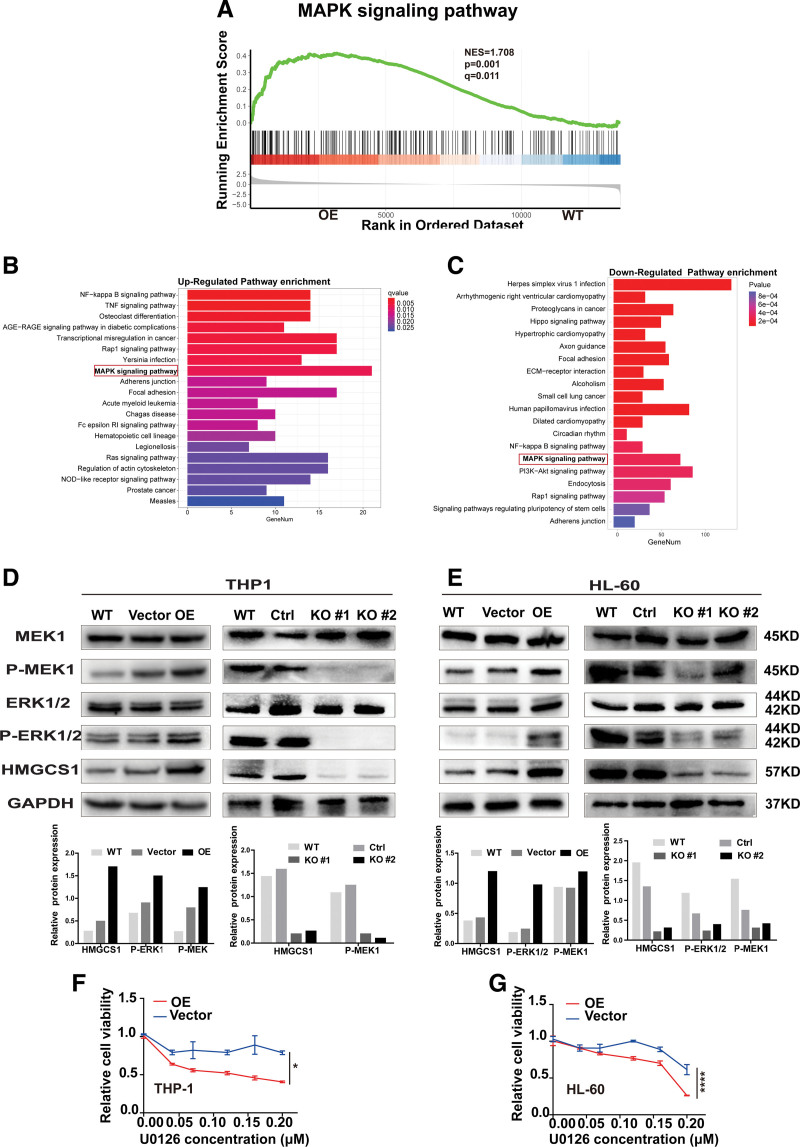
The activity of MAPK pathway in AML cells. (A) GSEA enrichment of MAPK pathway in THP-1 OE stable cells. (B–C) Upregulated and downregulated pathways in THP-1 OE (B) and KO (C) stable cells. (D–E) Western blot of MEK1, p-MEK1, ERK1/2, p-ERK1/2, HMGCS1 level in HMGCS1 OE/KO THP-1 and HL-60 cells and their control cell lines. The quantification of grayscale value was conducted with Image J. (F–G) Cell viability in OE cells treated with U0126. Cell viability was detected with CCK-8 kit 48 h after U0126 treatment. *P < .05, **P < .01, and ***P < .001. AGE-RAGE = advanced glycation end products-receptor for advanced glycation end products, AML = acute myeloid leukemia, ECM = extracellular matrix, ERK = extracellular signal-regulated protein kinase, GAPDH = glyceraldehyde-3-phosphate dehydrogenase, GSEA = gene set enrichment analysis, HMGCS1 = hydroxy-3-methylglutaryl coenzyme A synthase 1, KO = knockout, MAPK = mitogen-activated protein kinase, MEK = mitogen-activated protein kinase, NF = nuclear factor, NOD = non obese diabetes, OE = overexpression, PI3K-Akt = phosphatidylinositol-3-kinase-protein kinase B, TNF = tumor necrosis factor, WT = wild type.

To investigate the mechanism of how HMGCS1 regulates growth and sensitivity, we performed RNA-seq analysis using primary AML cells from patients and AML-HMGCS1 overexpressing and KO stable cell lines. In addition to primary AML cells, the MAPK pathway was identified as one of the pathways upregulated in GSEA analysis of THP-1 OE cells (**Fig. 4A**). Kyoto Encyclopedia of Genes and Genomes (KEGG) pathway analysis indicated that the MAPK pathway was upregulated in THP-1 OE cells and downregulated in THP-1 KO cells compared with their respective control cells (**Fig. 4B and C**). Western blot confirmed that p-MEK1 and p-ERK1/2 levels were significantly increased in OE cells while decreased in KO cells for both THP-1 and HL-60 cells (**Fig. 4D and E**). As expected, U0126, an inhibitor of MEK1, significantly reduced the cell growth rate of OE cells (**Fig. 4F and G**). Collectively, our data suggest that HMGCS1 upregulation is positively correlated with activation of the MAPK pathway in AML RR patients and HMGCS1 activates MAPK pathway in AML cell lines.

### 3.5. Inhibition of HMGCS1 with its inhibitor hymeglusin reduces the growth rate of AML cells and increases their drug sensitivity

**Figure 5.**
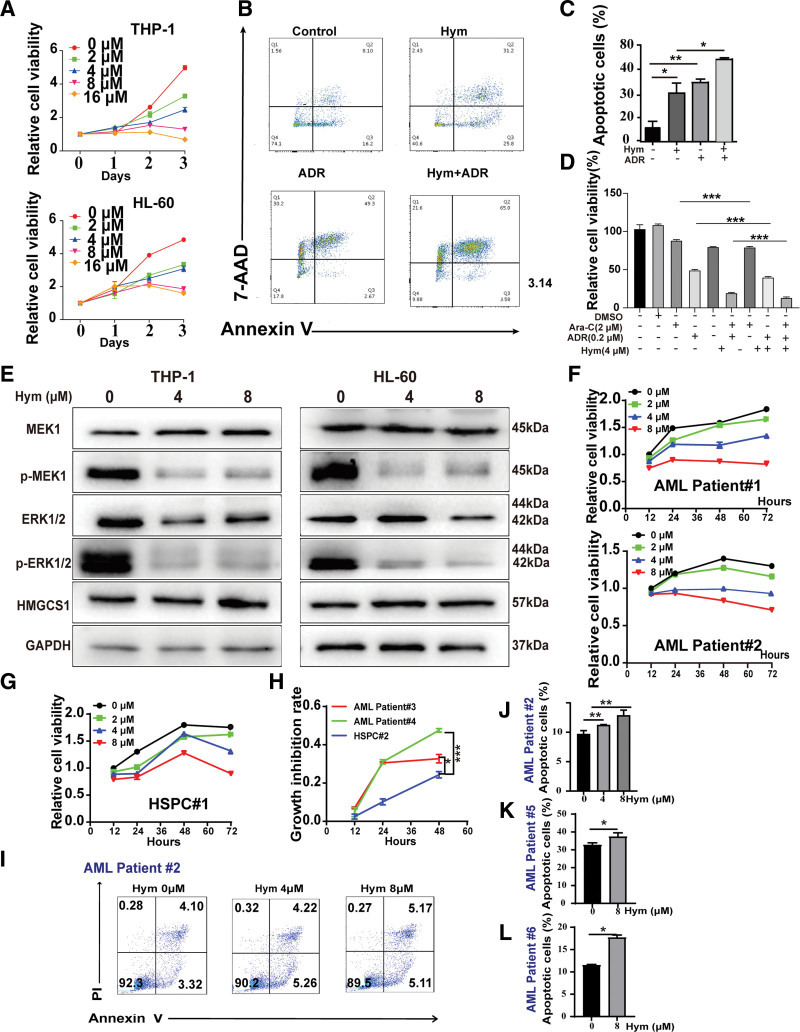
The effects of HMGCS1 inhibitor hymeglusin on AML cell. (A) Cell viability with the treatment of increasing concentration of hymeglusin in THP-1 and HL-60 cells. (B) Representative flow plots showing the apoptosis of THP-1 cells treated with Hym, ADR or both. Data were collected 48 h post treatment. (C) Statistical analysis of apoptotic THP-1 cells in (B). (D) Relative cell viability with combined treatment of Hym, ADR, and Ara-C. Cell viability was detected with CCK-8 kit 48 h after addition of drug treatment. (E) p-MEK1 and p-ERK1/2 level after Hym treatment in AML cells. Protein samples were collected 48 h after Hym treatment. (F) The relative cell viability in bone marrow cells of AML patients (G–H) and HSPCs from healthy donor (F–H) treated with different concentration of Hym (0, 2, 4, 8 μM) for 12, 24, and 48 h. (H) The growth inhibition rate in bone marrow cells of AML patients and HSPCs from healthy donor treated with Hym (4 μM) for 12, 24, and 48 h. (I) Representative plots of apoptosis analysis of bone marrow cells from patient #2 by flow cytometry. (J) Statistical analysis of apoptotic cells in (I). (K–L) Statistical analysis of apoptotic primary cells from patients #5 and #6 in (S5). *P < .05, **P < .01, and ***P < .001. AAD = 7-aminoactinomycin D, ADR = adriamycin, AML = acute myeloid leukemia, DMSO = dimethyl sulfoxide, ERK = extracellular signal-regulated protein kinase, GAPDH = glyceraldehyde-3-phosphate dehydrogenase, HMGCS1 = hydroxy-3-methylglutaryl coenzyme A synthase 1, Hym = hymeglusin, HSPC = hematopoietic stem and progenitor cell, MEK = mitogen-activated protein kinase.

Because HMGCS1 promotes cell growth and reduces drug sensitivity, we proposed that inhibition of HMGCS1 in AML cells could reduce AML cell growth and increase their drug sensitivity. To verify whether inhibition of HMGCS1 could induce the proposed effects, we investigated the effects of hymeglusin, a specific HMGCS1 inhibitor, in THP-1 and HL-60 cells. As expected, hymeglusin reduced the viability of both cell lines, similar to the effects of knocking out HMGCS1 (**Fig. 5A**). The 72-hour half maximal inhibitory concentration (IC50) was approximately 3.5 μM for THP-1 cells and 5.6 μM for HL-60 cells. The apoptosis level of THP-1 cells was significantly higher in the presence of hymeglusin than control, and it was further elevated when treated with the combination of hymeglusin and ADR (**Fig. 5B and C**). We also observed an increased cell cycle and apoptosis level for THP-1 cells when the HMGCS1 expression level was knocked down (Figures S4 and S5, http://links.lww.com/BS/A98). ADR and Ara-C are common chemotherapeutic drugs in the clinic. Interestingly, inhibition of the cell viability of THP-1 cells was observed when hymeglusin was added together with either ADR or Ara-C, and the most significant inhibition level was achieved when hymeglusin was added with Ara-C or with the combination of ADR and Ara-C (**Fig. 5D**). Consistent with results from the HMGCS1 KO cell lines, the MAPK pathway was suppressed after hymeglusin treatment in AML cells (**Fig. 5E**). http://links.lww.com/BS/A98 To explore whether inhibition of HMGCS1 with its inhibitor hymeglusin is feasible for AML treatment, we further examined the inhibitory effects of hymeglusin in primary bone marrow samples. As expected, the addition of hymeglusin effectively inhibited the growth of primary AML cells (**Fig. 5F** and Figure S6, http://links.lww.com/BS/A98). Notably, minimal effects on hematopoietic stem and progenitor cells (HSPCs) were observed when hymeglusin was used at low concentration (**Fig. 5G–H**). However, when used at high concentration, hymeglusin is also toxic to normal HSPCs. Therefore, the combination of hymeglusin with other chemotherapy drugs should be examined to minimize the cytotoxicity to normal HSPCs while eradicating AML blasts. Next, we tested whether inhibiting HMGCS1 with hymeglusin could induce the apoptosis of primary AML cells. Similar to the AML cell lines, hymeglusin also significantly increased the percentage of apoptotic cells in treated primary bone marrow cells (**Fig. 5I–L** and Figure S7, http://links.lww.com/BS/A98). Collectively, these data suggest that the HMGCS1 inhibitor hymeglusin can be used to inhibit growth and induce the apoptosis of AML cells.

## 4. DISCUSSION

In this study, we investigated and demonstrated the vital role of HMGCS1 in RR AML. We demonstrated that OE of the cholesterol synthesis upstream enzyme HMGCS1 is positively correlated with RR status and poor survival. Moreover, elevated HMGCS1 mRNA is associated with elevated serum TC and LDL levels. Cholesterol is an essential structural component of the cell membrane.^20^ It is deducible that HMGCS1 could promote leukemia cell growth by providing building blocks for cell proliferation. Abnormal cholesterol homeostasis is observed in both refractory leukemia patients^21^ and leukemia cells exposed to chemotherapy.^22,23^ It is widely accepted that cholesterol consumption is higher,^23,24^ and cholesterol synthesis is enhanced in most leukemia cells.^8,21^ These data explain the higher serum cholesterol/lipid levels in RR compared CR patients, probably due to enhanced de novo cholesterol synthesis contributed by an increased HMGCS1 expression level in AML cells.

Previous association between the MVA and MAPK pathways has been reported in AML. The well-known statin family drug HMGCR triggers apoptosis in AML by regulating several signaling pathways, including the Raf/MEK/ERK pathway. Exposure to the MEK1 inhibitor PD98059 sensitizes AML cells to low, physiologically achievable levels of lovastatin. In this study, we demonstrated that HMGCS1 promotes cell growth and reduces drug sensitivity. Moreover, our data prove that HMGCS1 is positively correlated with activation of the MAPK pathway. It is possible that HMGCS1 promotes cell growth and reduces drug sensitivity through activation of the MAPK pathway. Many of the intermediate metabolites (eg, FPP and GGPP) were produced during cholesterol synthesis, regulating the post-translational prenylation of a variety of proteins, which is vital to ensure the function of those proteins.^25^ Small GTPases, such as Ras, translocate to the cell membrane with the prerequisite of prenylation modification to become fully functional.^26,27^ It is likely that increases in the FPP and GGPP levels accompanied by high expression of HMGCS1 enhance Ras activity, leading to cascade activation of downstream MEK1 and ERK1/2 and finally uncontrolled cell proliferation. Further study of the expression level of HMGCS1 and metabolic intermediates of cholesterol synthesis with more chemoresistant AML cell lines will help reveal whether HMGCS1 increases cell growth and reduces drug sensitivity through subsequent activation of the MAPK pathway. Yet, it is possible that HMGCS1 exerts its role through pathways both dependent and independent on cholesterol synthesis. Rescue assays with WT, dominant negative, or constitutively active forms of HMGCS1 in HMGCS1 KO cell lines (only knock-down of HMGCS1 was achieved in this study) will help to better illustrate the mechanism of how HMGCS1 affects cell proliferation and drug sensitivity.

**Figure 6.**
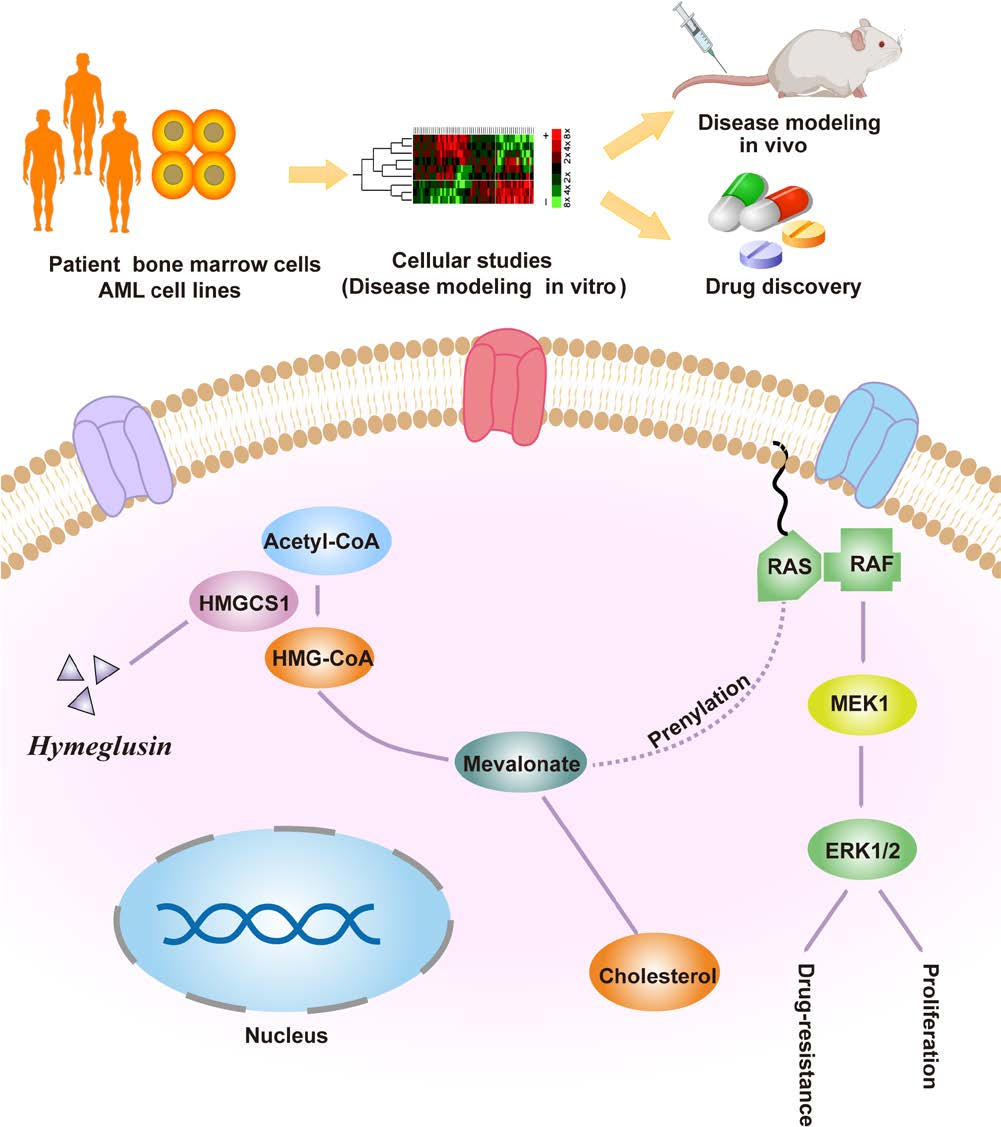
Working model for the role of HMGCS1. The schematic figure shows HMGCS1 plays dual functions in AML. HMGCS1 directly catalyzes the production of HMG-CoA, the latter is further transformed to FPP, GGPP, and cholesterol by multiple catalytic reactions. HMGCS1 promotes leukemia cell growth and drug resistance through the activation of MAPK signaling cascade and elevation of the cholesterol level. Hymeglusin could specifically inhibit the activity of HMGCS1 and may be a promising drug candidate in AML. AML = acute myeloid leukemia, ERK = extracellular signal-regulated protein kinase, FPP = farnesyl pyrophosphate, GGPP = geranylgeranyl pyrophosphate, HMG-CoA = 3-hydroxy-3-methylglutaryl coenzyme A, HMGCS1 = HMG-CoA synthase 1, MAPK = mitogen-activated protein kinase, MEK = mitogen-activated protein kinase, RAF = Raf, RAS = rat sarcoma.

Our findings also demonstrate that HMGCS1 mediates drug resistance. AML cells prevent against drug cytotoxicity by rapidly increasing the level of protective cholesterol, which is achieved by elevating the HMGCR and LDL-R mRNA levels.^8^ Our data further demonstrated that OE of HMGCS1, another gene upstream in the cholesterol synthesis pathway in leukemia cells could endow them with resistance to chemotherapeutic drugs. Moreover, we demonstrated that inhibition of HMGCS1 with its inhibitor hymeglusin reduces the growth of AML cells and increases their drug sensitivity. Notably, the addition of hymeglusin did not alter the HMGCS1 expression level. Hymeglusin was initially developed as an antibiotic, and later reported to have inhibitory effects on HMGCS1^28–30^ by specifically binding to the active site of Cys-129.^31^ Here, we demonstrated that hymeglusin could synergize with the chemotherapeutic drugs ADR and Ara-C to inhibit the growth of AML cell lines. These data suggest that hymeglusin is a promising drug candidate for treating AML (**Fig. 6**). However, when used at a high concentration, hymeglusin also inhibited the growth of normal HSPCs. Therefore, the combinatory regimen of hymeglusin and chemotherapeutic drugs should be explored to achieve inhibition of AML and minimize its toxicity toward normal HSPCs. We will further investigate the efficacy and safety of the combination of hymeglusin and other available inhibitors targeting the cholesterol synthesis pathway and standard anti-leukemia drugs to pursue highly efficient and precise treatment for AML.

We demonstrated that HMGCS1 promotes tumor growth in a nude mouse model. By employing this subcutaneous model, we observed that the tumor size and weight were significantly higher for THP-1/OE cells than control. For AML RR patients, the elevated level of HMGCS1 might also contribute to the growth of AML tumor cells. Further studies of intravenously established mouse models with AML cell lines that over-express HMGCS1 or AML RR primary cells will further illustrate the role of HMGCS1 in tumor growth. Administration of hymeglusin with chemotherapeutic drugs into these mouse models and subsequent analysis of changes in the expression levels of HMGCS1 and tumor burden would provide deeper insight into the efficacy of AML treatment with the combination of hymeglusin and common chemotherapy drugs. However, the cost of ****hymglusin as HMGCS1 inhibitor is high at the current stage for preclinical studies. Therefore, studies developing highly effective HMGCS1 inhibitors at low cost are definitely needed to achieve inhibition of HMGCS1 in preclinical models and clinical trials at affordable cost.

Collectively, the data presented here demonstrate that HMGCS1 promotes leukemia cell growth and drug resistance possibly through activation of the MAPK signaling cascade and elevation of the cholesterol level. HMGCS1 inhibitor may be a promising drug candidate for the treatment of AML. This work facilitates the understanding of cholesterol metabolism in AML and sheds light on the prospective translation of hymeglusin to the clinic.

## 5. CONCLUSIONS

In summary, our study demonstrates the important role of HMGCS1 in AML, and targeting this protein is promising for the treatment of RR AML.

## ACKNOWLEDGMENTS

This study was supported by the National Natural Science Foundation of China to H.Z. (Grant Nos. 81770184, 81970143, and 82270167) and L.Z. (Grant No. 81800174), the Talent Young Program of Guangdong Province (2021B1515020017), and the Leading Talents Program from The First Affiliated Hospital of Jinan University to H.Z.

We are very grateful to Dr. Christopher L. Robinson and Dr. Jinyong Wang for critically reading the manuscript. We acknowledge and appreciate our other colleagues for their valuable efforts and comments on this paper.

## ETHICAL APPROVAL

Studies involving human participants were reviewed and approved by the Institutional Research Ethics Committee of the Central South University-Xiangya Hospital (approval number: 201912530). All animal experiments were approved by the Institutional Animal Care and Use Committee of Central South University-Xiangya Hospital (IACUC-CSU-XH) and performed according to the approved protocols by IACUC-CSU-XH. All efforts were made to minimize the suffering of the included animals.

## AUTHOR CONTRIBUTIONS

H.Z. designed the project; C.Z., J.L., X.S., L.Z., H.Z., H.L., and P.F. performed the experiments; C.Z., J.L., X.S., L.Z., Q. H., and H.Z. analyzed the data; C.Z., J.L., J.D., L.Z., and H.Z. wrote and revised the manuscript; all authors have read and H.Z. approved the final submitted manuscript.

## Supplementary Material


