# Supplementary material for: Targeting HMGCS1 restores chemotherapy sensitivity in acute myeloid leukemia
Source: Blood Sci. 2024 Jul 10;6(3):e00192. doi: 10.1097/BS9.0000000000000192 (PMC11239175; doi:10.1097/BS9.0000000000000192)

Supplementary figures and figure legends

Figure S1. (A)The serum TG level in CR and RR patients without clinical usage of lipid lowering agents. (B) HMGCS1 mRNA level in AML WT cell lines (HL-60) and HL-60/ADR cells (ADR resistant HL-60 cells). (C) Protein level of HMGCS1 in HL-60 and HL-60/ADR cells detected by Western blot.(D)Protein level of HMGCS1 in THP-1 cells treated with ADR for 48h and detected by Western blot. (E)HMGCS1 mRNA level in THP-1 cells treated with ADR for 48h.

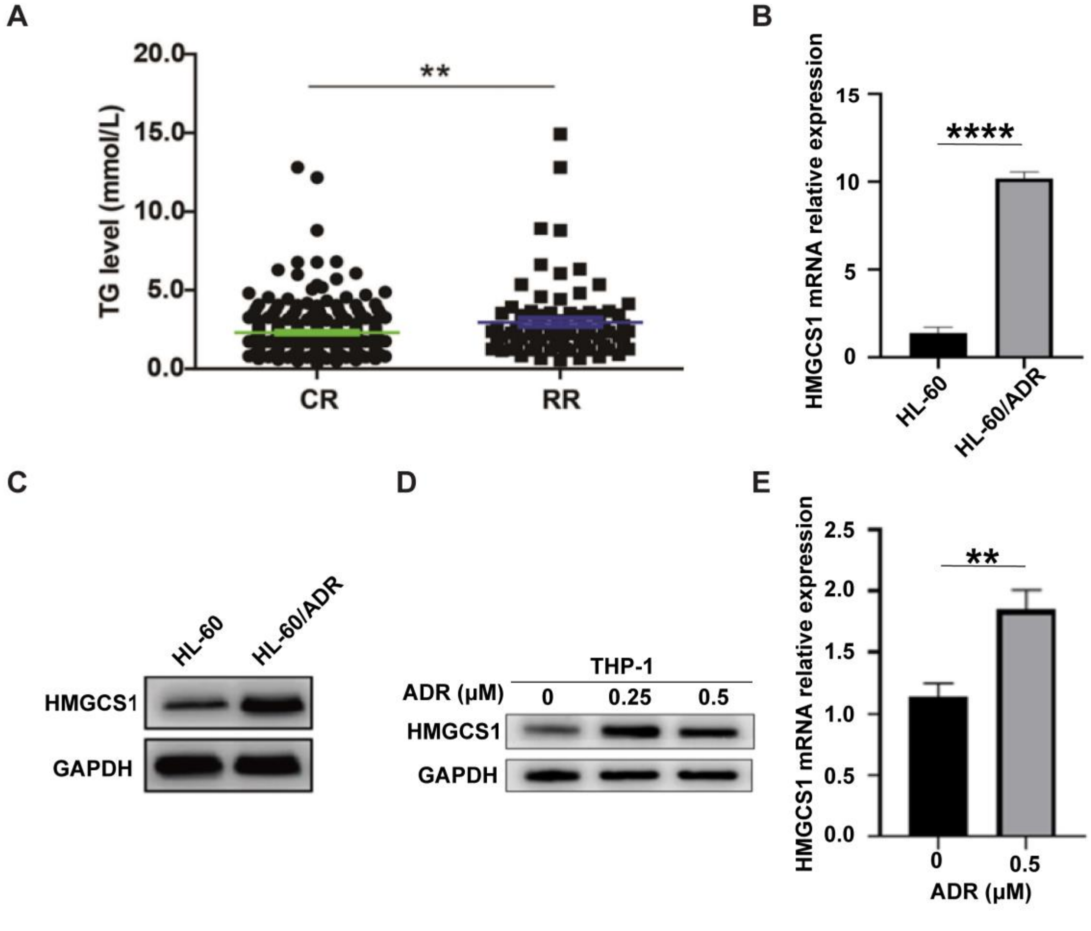

Figure S2. Validation of HMGCS1 KO and OE stable cells in AML cell lines. (A) Transcript expression of HMGCS1 in WT, Ctrl, KO (KO #1 and KO #2), Vector, and OE THP-1 cells. (B) The transcript expression of

HMGCS1 in WT, Ctrl, KO (KO #1 and KO #2), Vector, and OE HL-60 cells.

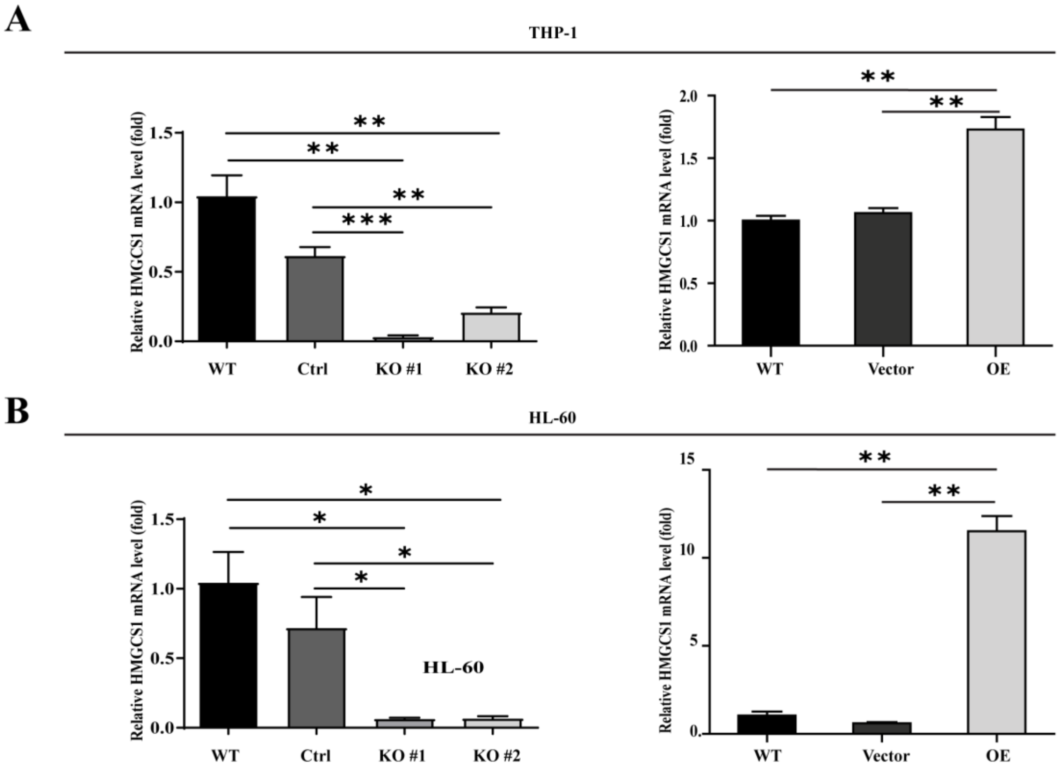

Figure S3. The effect of HMGCS1 KO in a xenograft nude mouse model. (A, B) Mouse weights (A) and growth rates (B) of tumors harvested from xenograft mice injected with THP-1 Ctrl/KO #2 cells (n=5 for each group). (C, D) Tumor image (C) and weight (D) derived from Ctrl/KO #2 cells.

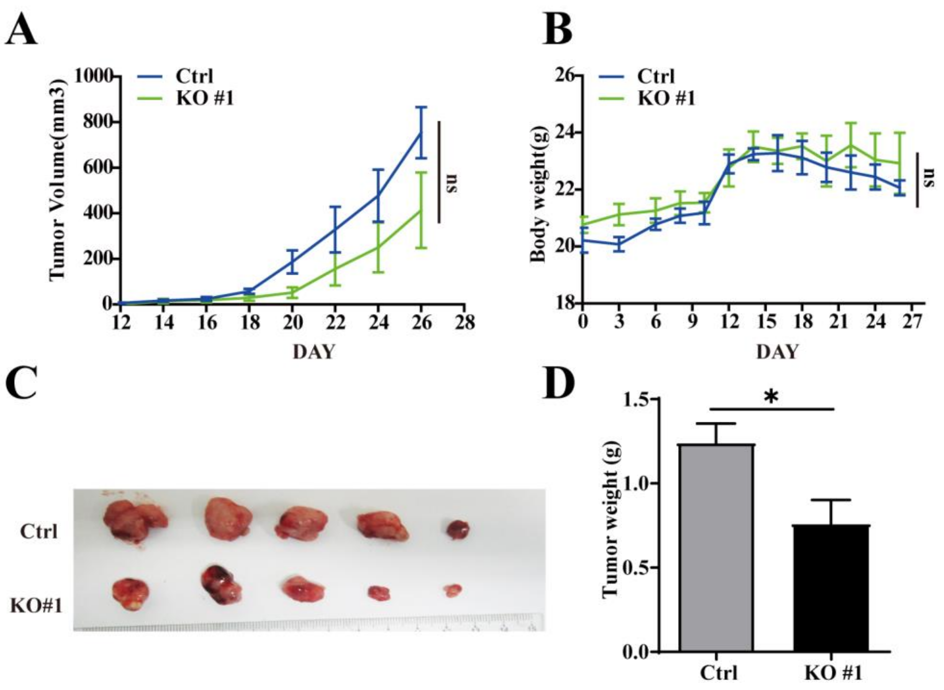

Figure S4. HMGCS1 affects the cell cycle and drug sensitivity of THP-1 cells. (A) Representative cell cycle analysis of WT/KO #2/OE stable cells and WT cells treated with hymeglusin (left). (B) The right part shows the quantification of Freq. S (%).

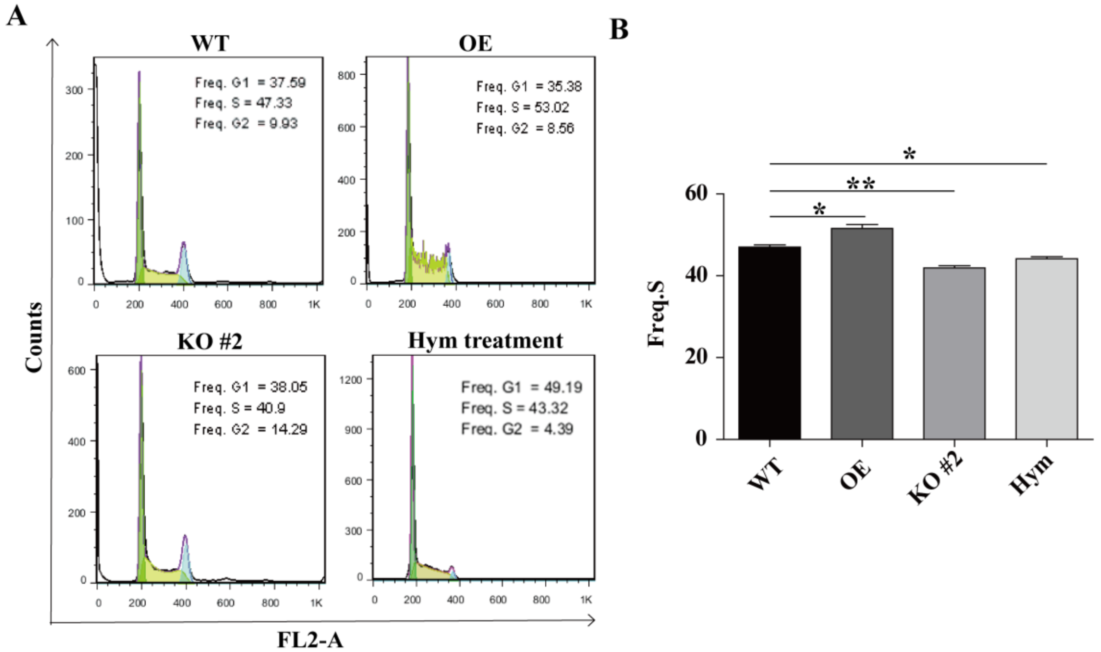

Figure S5. Inhibiting HMGCS1 enhances the ADR sensitivity of THP-1 cells. (A) Representative scatter plots showing the apoptosis rate of WT, KO#2, OE cells treated with ADR. (B) Quantification of the apoptosis rate in (A).

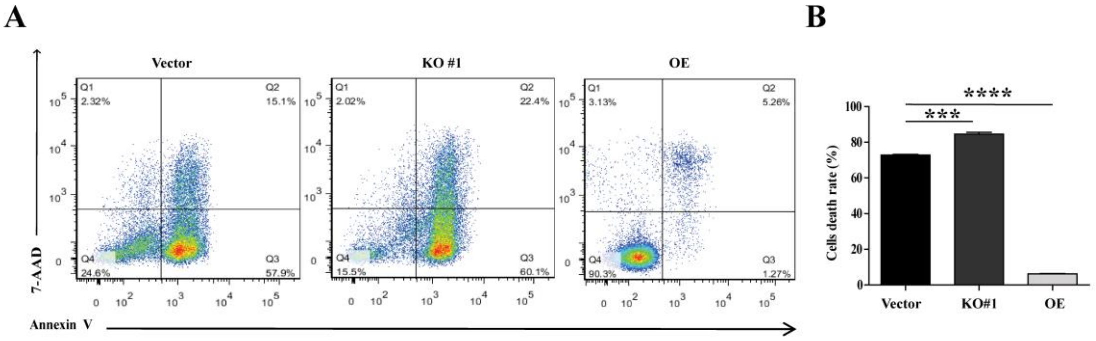

Figure S6. Effects of inhibition of HMGCS1 with 4 μM hymeglusin in primary AML cells. (A) The growth inhibition rate of AML Patient#3 and AML Patient#4 cells treated with hymeglusin. (B) Transcript expression of HMGCS1 in AML Patient#3 and AML Patient#4. Hym, hymeglusin.

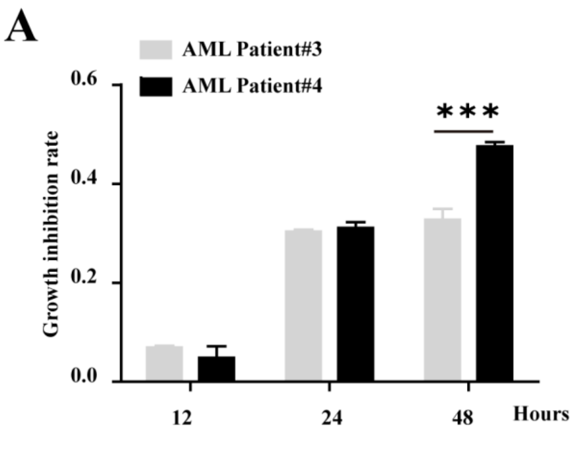

Figure S7. Representative plots of apoptosis analysis of bone marrow cells from patient#5 and #6 by flow cytometry. Primary bone marrow cells were treated with increasing concentrations of Hym for 48 h and used for Annexin V/PI staining.

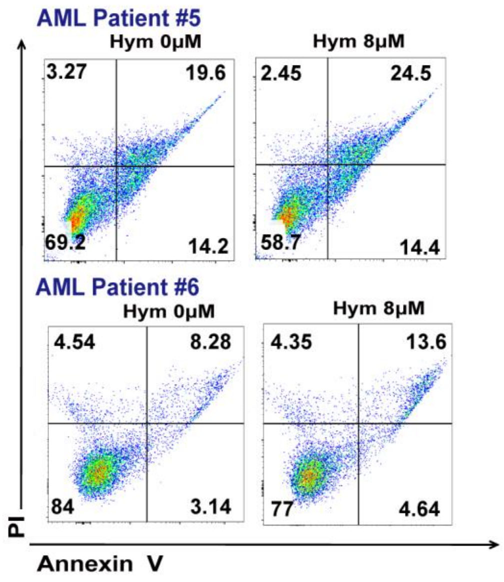

Supplement: Supplementary file 1 [file bs9-6-e00192-s001.pdf]
